# Therapeutic Efficacy of Intravesical Platelet-Rich Plasma Injections for Interstitial Cystitis/Bladder Pain Syndrome—A Comparative Study of Different Injection Number, Additives and Concentrations

**DOI:** 10.3389/fphar.2022.853776

**Published:** 2022-03-22

**Authors:** Yuan-Hong Jiang, Jia-Fong Jhang, Teng-Yi Lin, Han-Chen Ho, Yung-Hsiang Hsu, Hann-Chorng Kuo

**Affiliations:** ^1^ Department of Urology, Hualien Tzu Chi Hospital, Buddhist Tzu Chi Medical Foundation, Tzu Chi University, Hualien, Taiwan; ^2^ Department Laboratory Medicine, Hualien Tzu Chi Hospital, Buddhist Tzu Chi Medical Foundation, Hualien, Taiwan; ^3^ Department of Anatomy, Tzu Chi University, Hualien, Taiwan; ^4^ Department of Pathology, Hualien Tzu Chi Hospital, Buddhist Tzu Chi Medical Foundation, Tzu Chi University, Hualien, Taiwan

**Keywords:** platelet, therapeutic results, bladder pain, interstitial cystitis, bladder pain syndrome

## Abstract

**Purpose:** Intravesical platelet-rich plasma (PRP) injections have been demonstrated effective in relieving symptoms among patients with interstitial cystitis/bladder pain syndrome (IC/BPS). This study compared the clinical efficacy among different injection number, adding solution, and concentrations of PRP.

**Methods:** A total of 63 patients with IC/BPS were enrolled and randomly allocated to four subgroups who received single high-dose PRP (from 100 ml whole blood) plus 10 ml of normal saline or plasma injected over 20 or 40 sites. Patients were followed up at 1, 3, and 6 months for changes in the IC symptom index (ICSI) and problem index (ICPI), visual analog scale (VAS), global response assessment (GRA), and urodynamic parameters. Furthermore, we compared the clinical outcome with our previous study in a group of 55 IC/BPS patients who underwent four monthly low-dose PRP (from 50 ml whole blood) injections.

**Results:** The result of this study showed significant improvements in IC symptoms (ICSI 11.9 ± 4.4 *vs*. 10.2 ± 4.9, *p* = 0.009; ICPI 12.3 ± 3.4 *vs*. 10.6 ± 4.7, *p* = 0.003); VAS (5.46 ± 2.96 *vs*. 3.83 ± 3.1, *p* 0.000), and maximum flow rate (10.4 ± 4.9 *vs*. 17.1 ± 11.5 ml/s, *p* = 0.000) at 3 months after single high-dose PRP injection. However, no significant differences in therapeutic results were observed among subgroups, regardless of the added component or injecting site. The improvements of ICSI, ICPI, and GRA at 6 months were lower in comparison with the results of four low-dose PRP injections. All patients were free of dysuria, urinary retention, or urinary tract infection after PRP treatment.

**Conclusion:** Intravesical PRP injection is effective for IC/BPS. The addition of normal saline or plasma and injection site had no influence on therapeutic efficacy. However, the symptom improvement and GRA after a single high-dose PRP injection was lower than that after four low-dose PRP injections 6 months after the first treatment. Limitation of the study is lack of sham control group.

## Introduction

Interstitial cystitis/bladder pain syndrome (IC/BPS) is a clinical diagnosis based on symptoms including urinary frequency, urgency and bladder pain, pressure, and/or discomfort in the absence of other pathologic findings (Abrams et al., 2002; Hanno et al., 2015). The pathophysiology of IC/BPS involves urothelial dysfunction resulting in barrier defects, chronic inflammation, increased urothelial cell apoptosis, nociceptive receptor up-regulation, mast cell activation, and somatic functional syndrome (Keay, 2008; Parsons, 2011; Shie et al., 2012). With persistent suburothelial inflammation, the increased urothelial cell apoptosis and decreased cell proliferation cause impaired mucosal integrity and increased urothelial permeability, promoting causing bladder pain and irritation symptoms (Shie and Kuo, 2011). Hence, the pathophysiology of IC/BPS is likely associated with impaired urothelial progenitor cell regeneration upon trauma or infection.

Platelet-rich plasma (PRP) has been widely utilized as a therapeutic in orthopedics, dermatology, and ophthalmology (Etulain, 2018). PRP is rich in growth factors that promote cell proliferation, differentiation, and wound healing of defective epithelium (Mussano et al., 2016). PRP also secretes several pro- and anti-inflammatory cytokines that can activate a new inflammatory process and address previously unsolved inflammation, thereby eliminating neuropathic pain (Kuffler, 2013). Recent evidence has shown that platelets also act as a modulator in the process of tissue inflammation and regeneration (Etulain, 2018). PRP injections have been widely utilized for the treatment of osteoarthritis through the anti-inflammatory effects of platelet-related growth factors (Louis et al., 2018).

A recently completed clinical trial using four PRP intravesical injections for patients with IC/BPS refractory to conventional therapy in patients who completed the four injections treatment and follow-up visits showed that the Global Response Assessment (GRA) improved after the 1st PRP injection, with satisfaction persisting until the primary end-point (Jhang et al., 2019a). The success rate was 45, 52, 70, 70, and 67.5% after the 1^st^, 2^nd^, 3^rd^, 4^th^, and 3 months after the 4th PRP injection, respectively. The aforementioned study demonstrated that repeated intravesical injections of autologous PRP can safely and effectively decrease bladder pain, decrease frequency, and provide symptom improvement in patients with IC/BPS refractory to conventional therapy. Another study showed that repeated PRP injections promoted changes in urinary cytokines, growth factors, and functional proteins suggesting a significant decrease in urinary nerve growth factor, matrix metalloproteinase-13, and vascular endothelial growth factor levels after and that platelet derived growth factor PDGF-AB significantly increased 12 weeks after the first PRP treatment (Jiang et al., 2020a). Moreover, evidence has shown that the repeated intravesical PRP injections significantly decreased the Visual analog scale (VAS) pain score, frequency, and nocturia and improved GRA, thereby providing significant symptom improvement in patients with IC/BPS while appearing to be safe (Jhang et al., 2019b).

Although PRP has been widely utilized in the treatment of different local inflammatory diseases, no current standard preparation and protocol for obtaining the optimal PRP solution for treatment of IC/BPS have been available. An initial soft spin to obtain the platelet containing plasma, followed by a hard spin to obtain the platelet pellets has been the standard procedure for obtaining a PRP solution (Amable et al., 2013). However, the optimal injection number, added components, and plasma volume, which might influence therapeutic results, have not been established. A recent investigation has shown that adding normal saline (N/S) instead of platelet-poor plasma (PPP, which contains antiplatelet factors) to the platelet pellets could improve the therapeutic effects on would healing and angiogenesis in an animal model (Etulain et al., 2018). Thus, determining the best preparation and protocol for optimal therapeutic results is imperative for a novel PRP treatment for IC/BPS. The current study sought to compare the clinical efficacy of PRP according to different injection number, different additive, and treatment protocol (single high-dose PRP vs. four low-dose PRP injections) in the treatment of patients with IC/BPS.

## Materials and Methods

This study consisted of two parts: (1) comparison of therapeutic efficacy of intravesical PRP injection according to different regimen and injection sites and (2) comparison of therapeutic efficacy of intravesical PRP injections according to different treatment protocol.

All patients had been previously confirmed to have IC/BPS according to the NIDDK criteria (Hanno and Sant, 2001). Briefly, they had cardinal symptoms of frequency nocturia and bladder pain not relieved by conventional treatment for more than 6 months, and no lower urinary tract pathology could be identified (Homma et al., 2020). Further, all patients had glomerulations of bladder wall after cystoscopic hydrodistention (Yu et al., 2021). On enrolment, they were requested to keep a 3-day voiding diary prior to treatment to record functional bladder capacity (FBC) and the number of urinary frequency and nocturia. IC symptoms were assessed using the O’Leary-Sant score (OSS), including the IC symptom index (ICSI) and IC problem index (ICPI) (Lubeck et al., 2001). Pain scores were determined through patient self-assessment using a 10-unit VAS system. Videourodynamic study and potassium chloride (KCl, 0.4 M) sensitivity test (Parsons et al., 1998) were routinely performed to confirm that patients had a reduced cystometric bladder capacity and painful response to KCl test, and exclude the presence of detrusor overactivity and bladder outlet obstruction (Jhang et al., 2019a). All patients were informed of the possible complications associated with intravesical PRP injection, such as hematuria, micturition pain, difficult urination, transient urinary retention, or urinary tract infections (UTIs). Treatment outcomes were assessed using the GRA at 3 and 6 months after the first PRP injection (Wang et al., 2019).

This study was approved by the Research Ethics Committee of Hualien Tzu Chi Hospital and Buddhist Tzu Chi Medical Foundation (IRB108-21-A). Each patient was informed regarding the study rationale and procedures, and written informed consent was obtained before treatment. All methods used in this study were conducted in accordance with relevant guidelines and regulations.

### Part 1: Comparison of the Therapeutic Efficacy of a Single High-Dose PRP Intravesical Injection With Different Preparation and Injection Sites

The first part of this study prospectively enrolled a total of 63 patients who were randomized to the following subgroups according to different PRP preparation and injection sites. Patients who satisfied all eligibility criteria study entry received different intravesical injections of PRP at: (1) 20 sites with 10 ml of PRP in N/S, (2) 40 sites with 10 ml of PRP in N/S, (3) 20 sites with 10 ml PRP in PPP, and (4) 40 sites with 10 ml of PRP in PPP.

**FIGURE 1:**
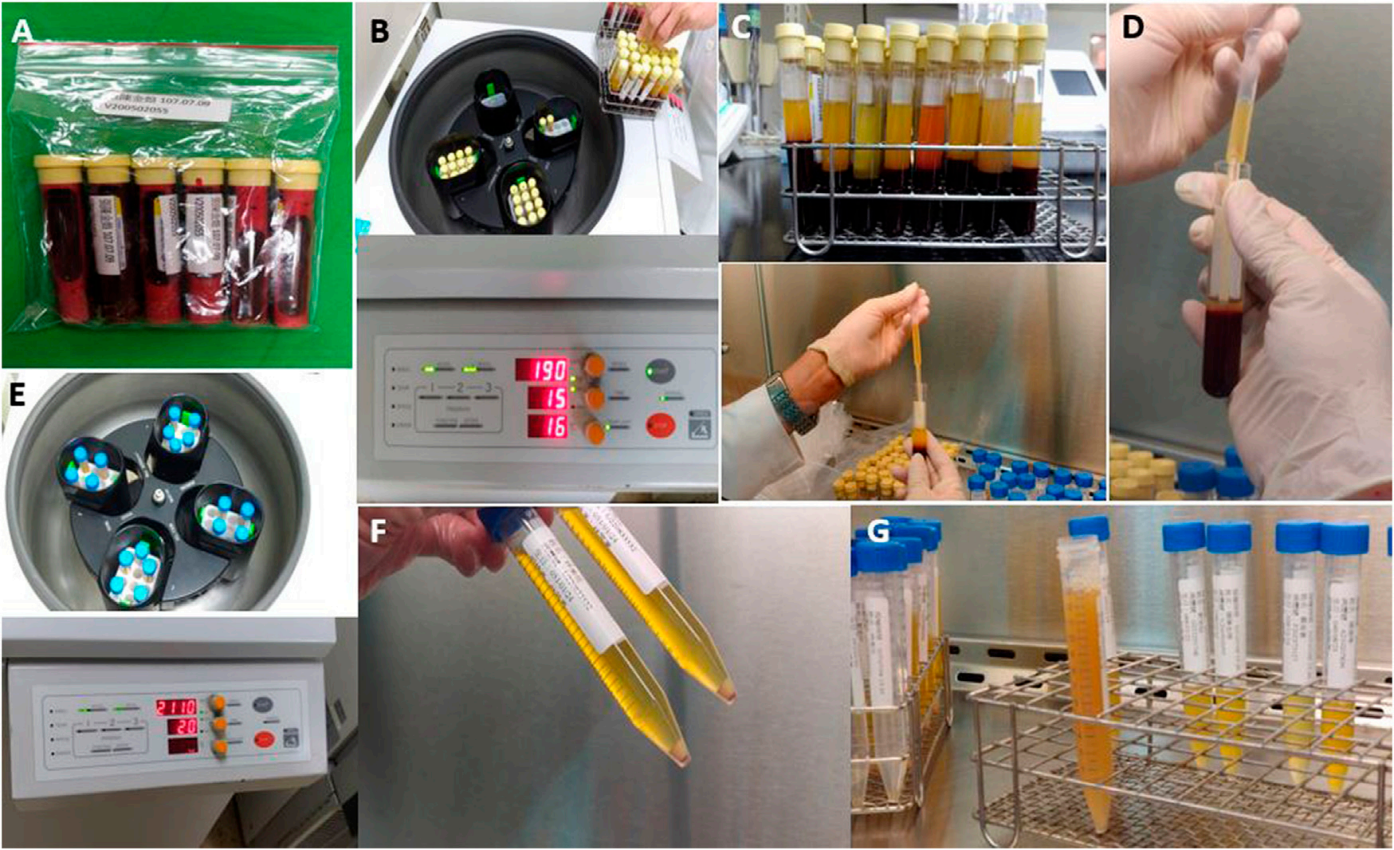
Preparation of platelet-rich plasma. **(A)** A total of 100 ml of whole blood was withdrawn; **(B)** Initial centrifugation of the blood with a soft spin (190 ×g, 20 min, <20°C); **(C)** The supernatant plasma containing platelets; **(D)** Supernatant plasma was transferred to another sterile tube without disturbing buffy coat; **(E)** The platelet containing plasma was further centrifuged via a hard spin (2000 ×g, 20 min, <20°C); **(F)** Platelet pellets were formed at the bottom of the tube; **(G)** The precipitate was platelet-rich plasma (PRP) and the upper part was platelet-poor plasma (PPP). After the PPP was removed, the platelet pellets were added to the required volume of PPP or normal saline to form sterile PRP for injection.

High-dose PRP was prepared according to the following procedures. A total of 100 ml of whole blood was withdrawn and sent to the central laboratory where the technologist initially centrifuged the blood with a soft spin (190 ×*g*, 20 min, <20°C). Thereafter, the supernatant plasma containing platelets was transferred to another sterile tube without disturbing buffy coat (without anticoagulant). The platelet containing plasma was further centrifuged via a hard spin (2000 ×*g*, 20 min, <20°C) (Jhang et al., 2019a). Platelet pellets were then formed at the bottom of the tube, with the lower third being PRP and the upper two thirds being PPP. After the PPP was removed, the platelet pellets were added to the PPP or N/S via gentle shaking of the tube to form 12 ml of sterile PRP. Thereafter, 1 ml of PRP was sent for culture, and another 1 ml for platelet count, leaving 10 ml PRP for intravescal injections (Figure 1).

The injection needle was inserted approximately 1 mm into the suburothelium at the posterior and lateral walls of the bladder at 20 or 40 sites, using a 23-gauge needle and rigid cystoscopic injection instrument (22 Fr, Richard Wolf, and Knittlingen, Germany). Cystoscopic hydrodistension was also performed under the intravesical pressure of 80 cmH_2_O, for activation of the injected platelets and evaluating the bladder glomerulation grade and measurement of maximal bladder capacity (Jhang et al., 2019a; Jhang et al., 2019b; Jiang et al., 2020a). After the PRP intravesical injections, a 14-Fr urethral Foley catheter was inserted for one night, with the patients being discharged on the next day. Because the PRP injection was performed under intravenous generous anesthesia, and multiple bladder injections were performed. It would be safe to observe the patient’s condition and keep an indwelling catheter overnight to avoid bleeding and urinary tract infection.

Oral antibiotics were prescribed for 3 days. Patients were followed up at the outpatient clinic 1, 3, and 6 months after PRP treatment. Data from the 3-day voiding diary and symptom inventory using the OSS score, as well as information on FBC, daily urinary frequency, nocturia episodes, and pain VAS, were recorded at baseline and 1, 3, and 6 months after PRP treatment. At 3 and 6 months after PRP injection, patients were questioned regarding their current bladder condition, after which a urodynamic study using the KCl test was performed.

The results of the voiding diary, urodynamic study, IC symptom score, and pain VAS were compared between baseline and primary end-point, as well as each secondary end-point. Successful results were assessed through self-reported improved GRA and pain VAS.

### Part 2: Comparison of the Therapeutic Efficacy of Intravesical PRP Injections Between Single High-Dose and Four Low-Dose PRP Treatment Protocol

This study collected our previous investigative data from 55 patients with IC/BPS who had received four monthly low-dose PRP injections for IC symptoms who were enrolled in our previous studies (Jhang et al., 2019a). The PRP was prepared using 50 ml of whole blood with the same centrifugation procedure as described above excepted for the addition of only PPP to form 10 ml of PRP for injection. All patients received 20 suburothelial injections of PRP solution, with each injection site receiving 0.5 ml of PRP. The injection technique was the same as described in the first part methodology. Only 20 injection sites were performed in all study patients. The data collection and follow-up protocol was also similar to that in the first part of the study.

Baseline demographics were compared between patients who received single high-dose PRP injection and those who received four monthly low-dose PRP injections. Changes from baseline in all measured variables 1, 3, and 6 months after PRP injections were then compared among the patients included in the first part of the study and among the different study groups in the second part of the study. Statistical comparisons between the groups were conducted using Pearson’s chi-square test or Fisher’s exact test for categorical variables and an independent *t*-test or ANOVA for continuous variables. A *p* value of <0.05 was considered statistically significant.

## Results

### Part 1: Comparison of the Therapeutic Efficacy of a Single High-Dose PRP Intravesical Injection With Different Preparation and Injection Sites

A total of 63 patients were enrolled in this prospective study, among whom 60 completed the visits at 3 and 6 months. The three patients who received the PRP injection and declined to follow-up visit because of having symptom improvement (*n* = 2) and no change (*n* = 1). Because there was no follow-up measurement, these patients were excluded from final analysis. Of these 60 patients, 15 received PRP in N/S at 20 sites, 14 received PRP in N/S at 40 sites, 15 received PRP in PPP at 20 sites, and 16 received PRP in PPP at 40 sites. No significant difference in age and gender were observed among the subgroups. Overall, improvement in VAS, ICSI, ICPI, and OSS were significant at 1, 3, and 6 months after PRP injection **(** Table 1). However, the significant increase in FBC was only observed at 1 month. GRA improved all at 1, 3, and 6 months. Moreover, a significant decrease in the PVR was observed after PRP injections.

**TABLE 1:** Changes in measured parameters from baseline to 1 and 3 months in patients treated with a single PRP injection.

	Baseline (*n* = 63)	1 M (*n* = 63)	3 M (*n* = 60)	6 M (*n* = 60)	P value (1 M)	P value (3 M)	P value (6 M)
VAS	5.46 ± 2.96	3.51 ± 2.67	3.83 ± 3.1	3.78 ± 3	0.000	0.000	0.000
ICSI	11.9 ± 4.4	9.91 ± 4.7	10.2 ± 4.9	10.3 ± 5	0.001	0.009	0.035
ICPI	12.3 ± 3.4	10.2 ± 4.4	10.6 ± 4.7	10.5 ± 4.3	0.000	0.003	0.003
OSS	24.2 ± 7.3	20.1 ± 8.8	20.7 ± 9.25	20.4 ± 9.3	0.000	0.003	0.004
Frequency	12.6 ± 5.9	11.9 ± 5.0	11.5 ± 3.4	11.6 ± 4.4	0.127	0.528	0.124
Nocturia	2.15 ± 1.4	1.91 ± 1.2	1.9 ± 1.1	1.9 ± 1.3	0.259	0.225	0.275
FBC(ml)	263 ± 95	301 ± 94.9	238 ± 103	277.7 ± 98.4	0.032	0.256	0.196
Qmax (ml/s)	10.4 ± 4.9	19.3 ± 12.1	17.1 ± 11.5	12.1 ± 7.0	0.000	0.000	0.512
Volume (ml)	220 ± 102	227 ± 126	235 ± 144	227 ± 105	0.736	0.548	0.582
PVR (mL)	47.3 ± 95.7	17.3 ± 25.5	25.4 ± 65.5	45.7 ± 85.1	0.027	0.025	0.045
GRA		1.26 ± 1.1	1.44 ± 1.2	1.5 ± 1.2			

PRP, platelet-rich plasma; VAS, visual analog scale; ICSI, interstitial cystitis symptom index; ICPI, interstitial cystitis problem index; OSS, O’Leary Sant symptom score; FBC, functional bladder capacity; Qmax, maximum flow rate; PVR, post-void residual; GRA, global response assessment.

The baseline clinical variables were not significantly different among four subgroups. After comparing the therapeutic efficacy between the four subgroups, we found no significant difference in all variables from baseline to each time point after PRP injections **(** Table 2). At 3 months, patients who received PRP in N/S at 20 sites and PRP in PPP at 40 sites showed a significantly improved GRA. At 6 months, 16 (55.5%) patients treated with PRP in N/S and 13 (41.9%) treated with PRP in PPP had a successful result (GRA ≥2). However, patients injected with PRP in PPP at 40 sites showed more remarkable improvement in ICS, ICPI, OSS, and VAS, although no significant difference was observed among subgroups in all variables. After comparing the therapeutic results between patients injected with PRP in N/S and PPP or between patients injecting with PRP at 20 and 40 sites, no significant difference in variables between groups was still observed at each time point.

**TABLE 2:** Comparison of the treatment results among IC/BPS patients treated with different PRP preparation and injection sites.

		PRP in N/S 10 ml at 20 sites (*n* = 15)	PRP in N/S 10 ml at 40 sites (*n* = 14)	PRP in PPP 10 ml at 20 sites (*n* = 15)	PRP in PPP 10 ml at 40 sites (*n* = 16)	*P* value
VAS	BL	6.54 ± 2.85	6.0 ± 2.95	4.46 ± 2.76	5.19 ± 2.86	0.383
	1 M	2.94 ± 2.86	4.73 ± 2.61	3.71 ± 2.92	3.07 ± 2.15	0.110*
	3 M	4.23 ± 3.0	5.08 ± 3.32	3.54 ± 3.26	2.81 ± 2.71	0.237*
	6 M	3.85 ± 3.1	4.91 ± 3.31	4.01 ± 3.22	2.71 ± 2.51	0.167*
ICSI	BL	10.6 ± 5.65	13.3 ± 3.39	11 ± 4.47	12.4 ± 3.33	0.271
	1 M	9.12 ± 5.16	11.5 ± 5.52	10.1 ± 3.29	9.53 ± 4.94	0.607*
	3 M	9.54 ± 5.33	11.3 ± 4.9	11.3 ± 4.55	8.88 ± 4.91	0.552*
	6 M	10.0 ± 6.10	11.3 ± 5.0	11.8 ± 4.10	8.60 ± 4.60	0.102*
ICPI	BL	11.4 ± 4.65	13 ± 2.34	11.7 ± 2.14	12.9 ± 3.26	0.528
	1 M	9.65 ± 4.32	11.6 ± 4.68	10.1 ± 3.32	10 ± 5.25	0.870*
	3 M	9.77 ± 5.37	12.9 ± 4.03	10.7 ± 4.25	9.31 ± 4.51	0.319*
	6 M	9.20 ± 4.80	12.9 ± 4.0	11.3 ± 3.10	9.10 ± 4.20	0.059*
OSS	BL	22 ± 10.12	26.3 ± 5.26	23.4 ± 4.75	24.69 ± 6.64	0.414
	1 M	18.8 ± 9.53	23 ± 9.85	20.1 ± 6.31	19.5 ± 10.1	0.566*
	3 M	19.3 ± 10.4	24.2 ± 8.65	22.0 ± 8.58	18.2 ± 9.11	0.547*
	6 M	19.2 ± 10.5	22.4 ± 10.7	23.2 ± 7.0	17.6 ± 8.50	0.252*
Frequency/ day	BL	11.3 ± 4.13	17.1 ± 7.83	11.5 ± 3.61	11.5 ± 6.84	0.456
	1 M	10.0 ± 3.53	14.0 ± 6.61	12.1 ± 4.39	11.9 ± 5.62	0.113*
	3 M	11.5 ± 4.12	15.1 ± 6.35	12.0 ± 3.54	12.1 ± 5.65	0.758*
	6 M	10.1 ± 2.90	12.4 ± 6.70	12.5 ± 4.50	11.2 ± 3.70	0.048*
Nocturia/ night	BL	1.64 ± 1.31	2.58 ± 1.14	2.54 ± 1.68	1.88 ± 1.22	0.488
	1 M	1.37 ± 0.93	2.35 ± 0.37	2.54 ± 1.46	1.44 ± 1.33	0.517*
	3 M	1.52 ± 1.11	2.43 ± 1.35	2.55 ± 1.64	1.67 ± 1.45	0.576*
	6 M	1.50 ± 1.40	2.20 ± 0.80	2.50 ± 1.60	1.60 ± 1.40	0.955*
FBC (ml)	BL	303 ± 133	252 ± 76.62	266 ± 91.1	234 ± 72.5	0.589
	1 M	315 ± 102	344 ± 84.4	274 ± 99.1	284 ± 96.8	0.129*
	3 M	309 ± 127	307 ± 83.5	280 ± 101	257 ± 88.9	0.375*
	6 M	249 ± 117	298 ± 106	272 ± 108	288 ± 89.9	0.362*
Qmax (ml/s)	BL	13 ± 3.94	11.4 ± 5.3	9.5 ± 4.82	8.77 ± 3.63	0.312
	1 M	19.5 ± 11.5	21.7 ± 17.1	16.3 ± 9.94	19.6 ± 10.6	0.531*
	3 M	19.2 ± 4.45	20.4 ± 15.5	12.5 ± 8.37	18.0 ± 12.2	0.231*
	6 M	13.5 ± 5.11	11.3 ± 4.32	12.7 ± 10.2	9.61 ± 6.30	0.791*
Volume (ml)	BL	234 ± 99.4	247 ± 109	175 ± 79.3	239 ± 110	0.711
	1 M	198 ± 91.6	218 ± 108	213 ± 118	271 ± 169	0.150*
	3 M	251 ± 32.5	246 ± 106	176 ± 129	277 ± 191	0.752*
	6 M	258 ± 120	263 ± 81.9	170 ± 74.9	236 ± 123	0.387*
PVR (ml)	BL	78.8 ± 123	72.7 ± 122	86.7 ± 135	36.2 ± 83.6	0.652
	1 M	17.1 ± 15.9	6.4 ± 9.47	32.3 ± 40.2	13.5 ± 22.8	0.349*
	3 M	61.8 ± 137	12.6 ± 17.9	30.7 ± 54.3	8.82 ± 7.77	0.278*
	6 M	27.9 ± 50.9	12.9 ± 12.9	41.8 ± 30.4	37.5 ± 74.9	0.842*
GRA	1 M	1.59 ± 1.0	1.27 ± 1.74	0.93 ± 0.73	1.20 ± 1.01	0.450
	3 M	2.07 ± 0.99	1.25 ± 1.29	1.0 ± 1.41	1.40 ± 0.91	0.107
	6 M	2.1 ± 1.0	1.41 ± 1.30	0.9 ± 0.43	1.40 ± 0.20	0.126
GRA ≥2 (n, %)	1 M	10 (66.7%)	4 (28.6%)	3 (20%)	6 (37.5%)	0.129
	3 M	10 (66.7%)	6 (42.9%)	5 (33.3%)	8 (50%)	0.533
	6 M	9 (60%)	7 (50%)	5 (33.3%)	8 (50%)	0.699

*p values, statistics of variable between each time point and baseline.

PRP, platelet-rich plasma; VAS, visual analog scale; ICSI, interstitial cystitis symptom index; ICPI, interstitial cystitis problem index; OSS, O’Leary Sant symptom score; FBC, functional bladder capacity; Qmax, maximum flow rate; PVR, post-void residual; GRA, global response assessment.

### Part 2: Comparison of the Therapeutic Efficacy of Intravesical PRP Injections Between Single High-Dose and Four Low-Dose PRP Treatment Protocol

A total of 55 patients with IC/BPS were enrolled in our previous clinical trials on low-dose PRP (Jhang et al., 2019a). Among them, 52 were available for the 3- and 6-months visits. Only 60 patients receiving high-dose PRP enrolled in the first part of this study were included in the second part for comparison of treatment outcome. Table 3 shows the baseline parameters between both study groups. The comparison points between two study groups were set at baseline (first PRP injection), 1 month, 3 months (3 months after single high-dose PRP and before 4^th^ low-dose PRP), and 6 months (6 months after single high-dose PRP and 3 months after 4^th^ low-dose PRP).

**TABLE 3:** Baseline parameters between patients with interstitial cystitis/bladder pain syndrome receiving different platelet-rich plasma injection protocols.

	Low-dose PRP x4 (*n* = 52)	High-dose PRP x 1 (*n* = 60)	*p* value
IC symptom scores			
VAS	4.1 ± 3.1	5.4 ± 2.9	0.024
ICSI	10.2 ± 4.9	12 ± 3.9	0.030
ICPI	10.6 ± 4	12.4 ± 3.2	0.007
OSS	20.4 ± 8.8	24.4 ± 6.8	0.006
Voiding diary variables			
Frequency/day	13.1 ± 7.3	12.4 ± 5.9	0.610
Nocturia/night	2.5 ± 1.3	2.2 ± 1.4	0.450
FBC (ml)	298.7 ± 131.7	303.2 ± 149.1	0.878
Urodynamic parameter			
FSF (ml)	133.3 ± 62.7	132.1 ± 45.1	0.901
FS (ml)	206.8 ± 89.7	208.5 ± 67.8	0.906
US (ml)	228.6 ± 100	234.3 ± 73.5	0.724
Compliance (mL/cmH_2_O)	68.7 ± 48.7	59.9 ± 48.3	0.331
CBC (ml)	267 ± 113.2	263.3 ± 78.4	0.659
Pdet (cmH_2_O)	20.9 ± 14.3	19 ± 10.6	0.423
Uroflowmetry parameter			
Qmax (m/s)	10.2 ± 5.6	10.2 ± 5.6	0.449
Volume (ml)	217.3 ± 109.8	217.9 ± 96.6	0.974
PVR (ml)	53.3 ± 123.2	52.2 ± 98.3	0.960
Cystoscopic HD			
MBC (ml)	671.4 ± 178.4	788.8 ± 181.1	0.001
Glomerulation	1.7 ± 0.9	1.5 ± 0.9	0.338
Hunner’s IC/ NHIC	4(7.7%)/48(92.3%)	5(7.6%)/61(92.4%)	0.981

FSF, first sensation of filling; FS, fullness sensation of bladder; US, urge sensation; CBC, cystometric bladder capacity; Pdet, detrusor pressure; Qmax, maximum flow rate; MBC, maximal bladder capacity; NHIC, non-Hunner’s IC; PRP, platelet-rich plasma.

**FIGURE 2:**
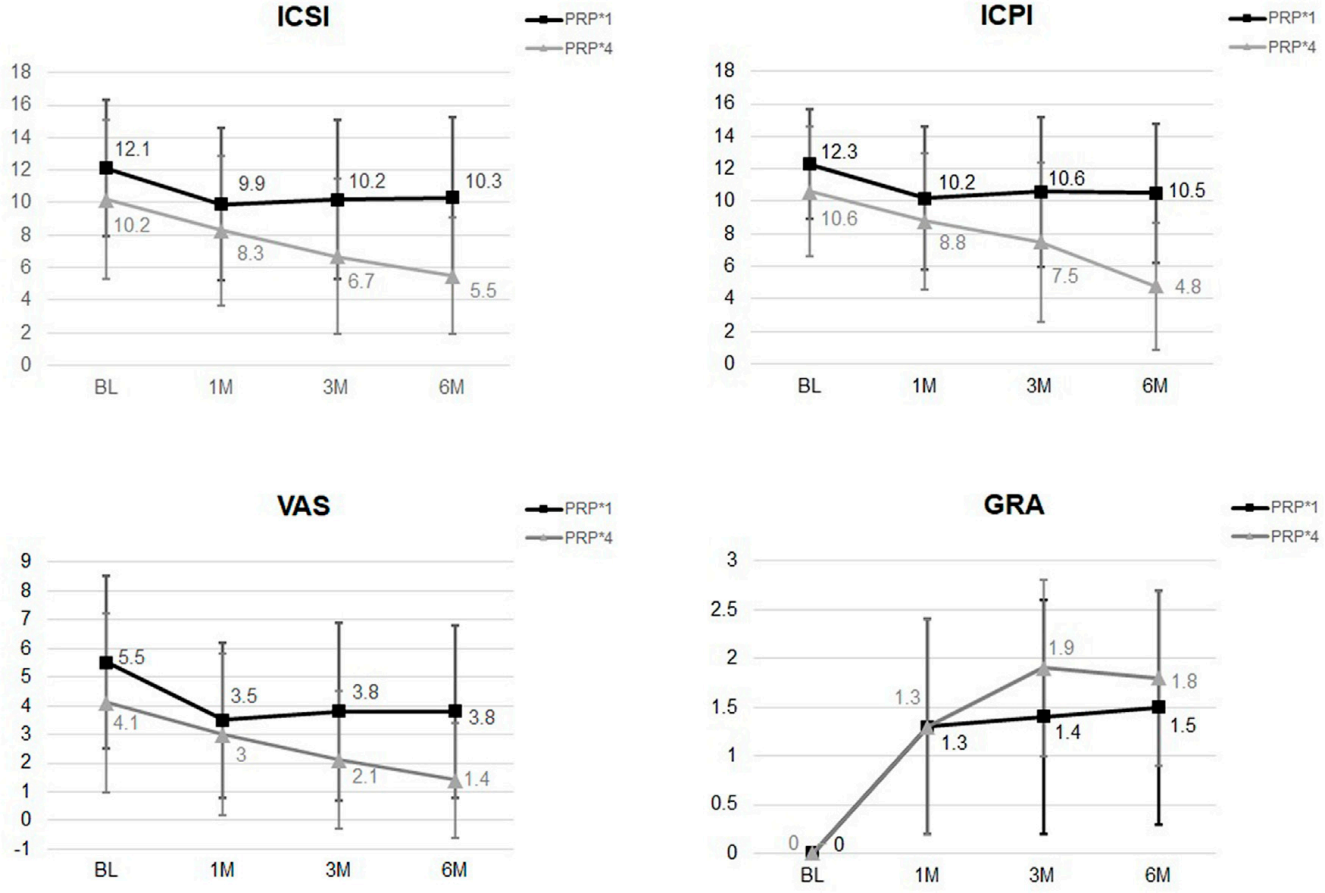
The changes of ICSI, ICPI, VAS, and GRA after different PRP injection protocols. Significant difference was noted in ICSI and ICPI from baseline to 6 months between groups.

After comparing the changes in IC symptoms from baseline to 1, 3, and 6 months after the first PRP injection, the only differences noted between patients treated with four low-dose PRP injections and single high-dose PRP injection at 6 months were in the ICSI and ICPI. **(** Table 4). Although VAS and OSS showed continued improvement at 6 moths in the low-dose PRP group, the difference between groups was not significant **(** Figure 2 **)**. The rates of patients with GRA ≥2 at 6 months was 67.5% (*n* = 27) and 48.3% (*n* = 29) in patients treated with four low-dose PRP and single high-dose PRP, respectively (*p* = 0.059). However, the rate of GRA ≥2 patients in the low-dose PRP group was significantly higher than that in high-dose PRP in PPP subgroups (*p* = 0.031), but not in high-dose PRP in PPP subgroups (*p* = 0.297). Patients receiving high-dose PRP in PPP at 20 sites had lowest rate of GRA ≥2 patients. (Table 2)

**TABLE 4:** Changes in measured IC symptom score parameters among treatment groups from baseline to end-points.

IC symptom scores		Low-dose PRP x4 (n = 52)	High-dose PRP × 1 (*n* = 60)	*p* value*
**VAS**	Baseline	4.1 ± 3.1	5.4 ± 2.9	
	1 M	3 ± 2.8	3.5 ± 2.7	0.082
	3 M	2.1 ± 2.4	3.8 ± 3.1	0.888
	6 M	1.4 ± 2	3.8 ± 3	0.394
**ICSI**	Baseline	10.2 ± 4.9	12 ± 3.9	
	1 M	8.3 ± 4.6	9.9 ± 4.7	0.729
	3 M	6.7 ± 4.8	10.2 ± 4.9	0.147
	6 M	5.5 ± 3.6	10.3 ± 5	0.038
**ICPI**	Baseline	10.6 ± 4	12.4 ± 3.2	
	1 M	8.8 ± 4.2	10.2 ± 4.4	0.541
	3 M	7.5 ± 4.9	10.6 ± 4.6	0.218
	6 M	4.8 ± 3.9	10.5 ± 4.3	0.000
**OSS**	Baseline	20.4 ± 8.8	24.4 ± 6.8	
	1 M	17.3 ± 8.4	20.1 ± 8.8	0.383
	3 M	13.6 ± 9.3	20.7 ± 9.2	0.082
	6 M	10.3 ± 7.1	19.0 ± 10.5	0.062

*p values, statistics between two groups in variable between each time point and baseline.

IC, interstitial cystitis; ICSI, IC symptom index; ICPI, IC problem index; OSS, O’Leary Sant symptom score; PRP, platelet-rich plasma.

The changes in voiding diary variables and uroflowmetry parameters also showed no significant difference between patients treated with low- and high-dose PRP. FBC was significantly increased in patients treated with low-dose PRP, although the difference was not significant **(** Table 5). Changes in urodynamic parameters from baseline to 6 months revealed that patients treated with low-dose PRP had significantly increased CBC. Interestingly, however, patients treated with high-dose PRP at the follow-up video urodynamic study had a decreased incidence of a positive KCl test **(** Table 6).

**TABLE 5:** Changes in voiding diary and uroflowmetry parameters among treatment groups from baseline to end-points.

		Low-dose PRP x4 (*n* = 52)	High-dose PRP× 1 (*n* = 60)	*p* value*
Voiding diary				
Frequency/day	Baseline	13.1 ± 7.3	12.4 ± 5.9	
	1 M	11.1 ± 5.1	11.8 ± 5.0	0.118
	3 M	10.4 ± 4.3	11.5 ± 3.4	0.528
	6 M	9.9 ± 3.4	11.6 ± 4.8	0.220
Nocturia/night	Baseline	2.5 ± 1.3	2.2 ± 1.4	
	1 M	2.5 ± 1.2	2.0 ± 1.2	0.402
	3 M	1.9 ± 1.1	1.9 ± 1.1	0.125
	6 M	2.0 ± 1.4	1.9 ± 1.2	0.255
FBC (ml)	Baseline	298.7 ± 131.7	303.2 ± 149.1	
	1 M	313.1 ± 113.6	298.8 ± 93.6	0.582
	3 M	329.1 ± 126.2	237.5 ± 102.8	0.356
	6 M	336.3 ± 103.2	287.7 ± 94.2	0.876
Uroflowmetry				
Qmax (m/s)	Baseline	10.2 ± 5.6	10.2 ± 5.6	
	1 M	17.4 ± 10.2	19.4 ± 11.9	0.594
	3 M	11.4 ± 5.4	17.3 ± 11.5	0.004
	6 M	11.3 ± 5.2	12.1 ± 7.0	0.780
Volume (ml)	Baseline	217.3 ± 109.8	217.9 ± 96.6	
	1 M	220.4 ± 129.7	233.4 ± 127.9	0.926
	3 M	229 ± 127.7	238.5 ± 143.8	0.822
	6 M	240.5 ± 120.7	226.6 ± 104.6	0.590
PVR (ml)	Baseline	53.3 ± 123.2	52.2 ± 98.3	
	1 M	25.1 ± 29.4	17.3 ± 25.5	0.631
	3 M	46.5 ± 90.9	25.4 ± 65.5	0.311
	6 M	45.9 ± 91.1	45.7 ± 86.1	0.445

*p values, statistics between two groups in variable between each time point and baseline.

FBC, function al bladder capacity; Qmax, maximum flow rate; PVR, post-void residual; PRP, platelet-rich plasma.

**TABLE 6:** Changes in urodynamic parameters among treatment groups from baseline to end-points.

Urodynamic parameters		Low dose PRP x4 (*n* = 52)	High dose PRP× 1 (*n* = 60)	*p* value*
FSF (ml)	Baseline	137.4 ± 61.4	125.7 ± 46.6	0.086
	6 M	129.5 ± 60.7	141.1 ± 53.8	
FS (ml)	Baseline	213.6 ± 86.5	203.9 ± 72.5	0.258
	6 M	200.7 ± 85.1	209.1 ± 74.3	
US (ml)	Baseline	233.4 ± 95.9	225.4 ± 75.7	0.148
	6 M	220.3 ± 92.3	236.2 ± 89.2	
Compliance (mL/cmH_2_O)	Baseline	69.4 ± 49.2	61 ± 55.7	0.427
	6 M	60.3 ± 51.8	62.9 ± 36.7	
CBC (ml)	Baseline	277.1 ± 112.6	260 ± 98.5	0.006
	6 M	284.2 ± 115.2	184.3 ± 144	
Pdet (cmH_2_O)	Baseline	21.2 ± 14.5	18.9 ± 11.8	0.617
	6 M	19 ± 10.4	18.1 ± 10.5	
Positive KCl (%)	Baseline	36 (69.2%)	53 (88.3%)	0.000
	6 M	25 (48.1%)	15 (25%)	

*p values, statistics between two groups in variable between 6 M and baseline.

FSF, first sensation of filling; FS, fullness sensation of bladder; US, urge sensation; CBC, cystometric bladder capacity; Pdet, detrusor pressure; KCl, potassium chloride; PRP, platelet-rich plasma.

## Discussion

This study demonstrated that either a single high-dose PRP injection or four low-dose PRP injections can improve IC symptoms after treatment. Although a single high-dose PRP injection can promote symptom improvement up to 6 months, the amplitude of symptomatic improvement was significantly lower compared to patients treated with four consecutive low-dose PRP injections. Using N/S or PPP or injecting PRP at 20 or 40 sites in the urinary bladder had no influence on the outcomes of single high-dose PRP treatment. But patients with high-dose PRP in PPP had a lower GRA compared with those with low-dose PRP treatment. No adverse events related to PRP injections, such as UTIs or acute urinary retention, occurred throughout the current study.

PRP has been previously used as regenerative medicine therapy in numerous medical applications (Kuffler, 2013; Etulain, 2018; Louis et al., 2018). PRP is rich in several types of growth factors, which increase the rate of proliferation and differentiation of injured tissue cells and repair tissue defects, thereby promoting early wound healing (Mussano et al., 2016)**.** PRP also secrets several types of cytokines that can initiate a new inflammatory process and facilitate the resolution of the unsolved inflammation, thereby eliminating the neurogenic pain caused by the previous inflammation (Kuffler, 2013). The rationale for using PRP in treating IC/BPS is the ease of obtaining material, concentration of PRP, and delivery of bioactive molecules into the bladder wall (Mussano et al., 2016). The platelet-related growth factors can also promote tissue angiogenesis, increase blood flow, and improve oxygenation in the wound (Roy et al., 2011).

IC/BPS is a condition characterized by inflammation and urothelial defects. Bladder dysfunction in IC/BPS can be attributed to an unresolved wound healing process and the defective urothelium, which induce bladder pain (Jhang et al., 2021a). The pathophysiology of IC/BPS might be associated with impaired regenerative ability of the urothelial cells. Thus, improving the progenitor cell regeneration could perhaps rebuild the bladder urothelial barrier and eliminate IC/BPS symptoms (Ke et al., 2019).

PRP administration into the bladder urothelium could initiate and complete the wound healing process, produce new inflammation, promote neurogenic pain relief. Activated PRP might induce locally new inflammation, which could override the unsolved inflammation, promote the wound healing process, and increase tissue regeneration. Our pilot clinical trial showed that four repeated intravesical PRP injections can significantly improve IC symptoms and bladder conditions while apparently being safe (Jhang et al., 2019b). We have previously conducted preliminary clinical trials using multiple low-dose PRP injections at 20 sites to treat patients with IC/BPS. Notably, our results have shown that four PRP injections can significantly ameliorate IC symptoms and decrease VAS pain scores (Jhang et al., 2019a). Repeated PRP injections have also been shown to significantly decrease urine cytokines (Jiang et al., 2020a).

Immunohistochemistry study showed that four repeated PRP injections increased the expression of the urothelial cell proliferation marker Sonic hedgehog protein and cytoskeleton marker CK*5*, as well as the expression of adhesive proteins E-cadherin and ZO-1, in patients with IC/BPS, indicating that PRP injections can increase progenitor cell proliferative activity and facilitate barrier protein expression. (Jhang et al., 2022). However, not all urothelial proteins show similar changes after PRP injection. Given that PRP contains concentrated platelet cells, therapeutic efficacy can only be achieved at the injection site and surrounding areas. Injecting PRP at the bladder wall or targeting sites where bladder inflammation is the most prominent might not cover the whole bladder wall. Thus, Injecting PRP across more sites might provide better therapeutic efficacy by covering most of the bladder.

Moreover, the plasma (PPP) in PRP preparation contains antiplatelet factors that might inhibit growth factor concentrations and reduce the therapeutic efficacy of activated PRP. One previous study also showed that adding N/S instead of PPP might promote better therapeutic results in terms of angiogenesis (Etulain et al., 2018). The first part of this study sought to compare the therapeutic efficacy between different PRP preparations by adding PPP or N/S on platelet pellets across different injection sites. Interestingly, our finding showed no significant differences among subgroups according to PRP preparation and injection site. However, we found patients with high-dose PRP in PPP, but not PRP in N/S, had a lower GRA at 6 months compared with those with low-dose PRP treatment. As such, our results suggested that N/S might be better for PRP preparations and that 20 PRP injection sites appear adequate for achieving significant therapeutic results. Adequate platelet cell counts and concentrations provide satisfactory therapeutic results; however, excessive injections might increase the risk of hematuria, UTI, and miction pain.

Although four intravesical injections of low-dose PRP have been demonstrated effective, such a treatment needs intravenous general anesthesia, which places tremendous burden on the patients. Four monthly injections might also promote complications associated with general anesthesia and bladder injections, such as sore throat, general weakness, miction pain, hematuria, and UTI. Thus, limiting the number of PRP injections to a single dose and doubling the dose might promote less morbidity in patients with IC/BPS after this invasive procedure. Therefore, the second part of the study we conducted was to determine whether a single high-dose PRP and four low-dose PRP injections promoted similar therapeutic efficacy. Notably, current study found reduced IC symptoms 6 months after the first PRP injection. Moreover, the ICSI and ICPI were significantly greater in patients receiving the four low-dose PRP injections than those receiving a single high-dose PRP injection group. Furthermore, patients receiving single high-dose PRP injection group showed no improvement in symptoms at 3 and 6 months, whereas those receiving the four-low dose PRP injection showed continues improvement. This could likely be attributed to the increased bladder area coverage following more PRP injections every month, which promoted better symptomatic improvement at 6 months. Based on the aforementioned result, a single high-dose PRP injection might have a limited therapeutic duration compared to four low-dose PRP injections. However, most patients with IC/BPS might not be able to tolerate the frequency of anesthesia and intravesical injections. Hence, identifying effective PRP injection programs with less treatment frequency and providing durable therapeutic efficacy is imperative.

Although PRP seems promising in treatment of IC/BPS refractory to conventional therapies, not all patients can benefit from this treatment. IC/BPS is a heterogeneous syndrome caused by different pathogenesis. We have previous found different phenotypes according to the presence of Hunner’s lesion or non-ulcer, and cystoscopic hydrodistention results (Yu et al., 2021), and histopathology findings (Jhang et al., 2021b). In severe forms of IC/BPS (grade 3 gomerulation and Hunner’s IC) decrease of urothelial cytoskeleton and cell proliferation protein expression are evident (Jhang et al., 2021a). With these data, it is crucial to identify a subset of IC patients who may benefit from PRP treatment. However, currently, we still cannot identify proper candidates for this treatment. Use of urinary biomarkers might be a potential tool to identify bladder IC and non-bladder IC (Jiang et al., 2020b).

The limitations of the current study include the small case number in the first part of the study and the consecutive study groups in the second part of the study. However, the number of patients has been adequate for the comparison between the four subgroups. Secondly, we did not have sham treatment group for comparison because the studies were very preliminary and the purpose was to prove its efficacy and safety. Moreover, the baseline IC symptoms (VAS, ICSI, ICPI, and OSS) are significantly greater in the high-dose PRP group than low-dose PRP group. This baseline bias of IC severity might result in a less favorable result in single high-dose PRP treatment. However, patients who received a single high-dose and four low-dose PRP injections were not randomly selected but consecutively enrolled. Further, patients of low-dose PRP group had a significantly smaller MBC, therefore, the baseline bias between study groups might be due to the factor of different research assistant, but not the true difference in the bladder condition. These studies were conducted to determine the appropriate PRP preparation and injection protocol. Based on the results of these studies, we suggest using low-dose PRP injections obtained from 50 ml whole blood and injecting at 20 sites every month to treat IC/BPS refractory to convention therapy.

## Conclusion

The current study demonstrated that intravesical PRP injections are effective in treating IC/BPS. Adding N/S or PPP and injecting at 20 or 40 sites showed no influence on therapeutic efficacy. However, 6 months after the first PRP injection, those receiving a single high-dose PRP injection had lower therapeutic GRA from baseline compared to those who received four low-dose PRP injections.

## Data Availability

The original contributions presented in the study are included in the article/Supplementary Material, further inquiries can be directed to the corresponding author.
